# Cardiorespiratory Fitness Is Associated with Selective Attention in Healthy Male High-School Students

**DOI:** 10.3389/fnhum.2017.00330

**Published:** 2017-06-28

**Authors:** Eivind Wengaard, Morten Kristoffersen, Anette Harris, Hilde Gundersen

**Affiliations:** ^1^Department of Sport and Physical Education, Western Norway University of Applied SciencesBergen, Norway; ^2^Department of Psychosocial Science, Faculty of Psychology, Institute of Psychosocial Science, University of BergenBergen, Norway

**Keywords:** aerobic fitness, VO_2_max, cognitive function, cue-target paradigm, attention

## Abstract

**Background**: Previous studies have shown associations of physical fitness and cognition in children and in younger and older adults. However, knowledge about associations in high-school adolescents and young adults is sparse. Thus, the aim of this study was to evaluate the association of physical fitness, measured as maximal oxygen uptake ($\overset{\cdot }{\text{V}}{\text{O}}_{2\max}$), muscle mass, weekly training, and cognitive function in the executive domains of selective attention and inhibitory control, in healthy male high-school students.

**Methods**: Fifty-four males (17.9 ± 0.9 years, 72 ± 11 kg and 182 ± 7 cm) completed a $\overset{\cdot }{\text{V}}{\text{O}}_{2\max}$ test, a body composition test and a visual cognitive task based on the Posner cue paradigm with three types of stimuli with different attentional demands (i.e., stimuli presentation following no cue, valid cue or invalid cue presentations). The task consisted of 336 target stimuli, where 56 (17%) of the target stimuli appeared without a cue (no cue), 224 (67%) appeared in the same rectangle as the cue (valid cue) and 56 (17%) appeared in the rectangle opposite to the cue (invalid cue). Mean reaction time (RT) and corresponding errors was calculated for each stimuli type. Total task duration was 9 min and 20 s In addition, relevant background information was obtained in a questionnaire.

**Results**: Linear mixed model analyses showed that higher $\overset{\cdot }{\text{V}}{\text{O}}_{2\max}$ was associated with faster RT for stimuli following invalid cue (Estimate = −2.69, SE = 1.03, *p* = 0.011), and for stimuli following valid cue (Estimate = −2.08, SE = 1.03, *p* = 0.048). There was no association of muscle mass and stimuli (*F* = 1.01, *p* = 0.397) or of weekly training and stimuli (*F* = 0.99, *p* = 0.405).

**Conclusion**: The results suggest that cardiorespiratory fitness is associated with cognitive performance in healthy male high-school students in the executive domains of selective attention.

## Introduction

In recent years, researchers have become increasingly interested in investigating possible impacts of physical activity and physical fitness on cognitive functions, and several studies have suggested that higher physical fitness levels are associated with cognitive benefits (Guiney and Machado, 2013; Dupuy et al., 2015; Cox et al., 2016).

The vast majority of research has been conducted with older adults (above 50 years), and shows positive associations of physical activity and cognitive function (Colcombe and Kramer, 2003), as well as a reduced risk of age-related cognitive decline (Paillard et al., 2015). Recently, a growing number of studies have shown positive associations between physical fitness and cognitive function in developing children and young adolescents (Hillman et al., 2009; Chaddock et al., 2011; Fedewa and Ahn, 2011; Esteban-Cornejo et al., 2015). Positive associations are also reported in young adults (Themanson and Hillman, 2006; Kamijo and Takeda, 2009), however with inconsistent findings (Scisco et al., 2008; Hayes et al., 2014). Knowledge about the associations in high-school adolescents and young adults are still sparse (Cox et al., 2016).

Given that executive functions develop throughout the childhood and decline with age (Friedman et al., 2009), the effects of physical fitness may be elusive in high-school students and in young adults where cognition is developmentally peaking (Hayes et al., 2014). As a result, specific and sensitive methods of measurement are required. In general, a large number of different methods of measurement have been employed in research in this field, as well as different definitions of cognitive function (Biddle and Asare, 2011). Dupuy et al. (2015) have stressed that a major weakness in many studies is the fact that fitness is often measured by subjective self-reports or through submaximal tests. An objective measure of cardiorespiratory fitness, $\overset{\cdot }{\text{V}}{\text{O}}_{2\max}$, was therefore chosen in the present study, in addition to self-reported weekly physical activity and objectively measured muscle mass.

In general, previous research indicates that the association of physical fitness and cognitive function is stronger for tasks requiring higher level of executive functioning (Colcombe and Kramer, 2003; Hillman et al., 2009; Pontifex et al., 2011; Guiney and Machado, 2013), particularly those tasks related to different aspects of attentional ability such as inhibition and task switching (Themanson et al., 2008; Guiney and Machado, 2013; Buckley et al., 2014). A cognitive task with different attentional load was therefore chosen in the present study to investigate the association of physical fitness and cognitive load.

In terms of age, the high-students in our study came close to what could be categorized as young adulthood, thus the study adds to previous work done on subjects in late adolescence or young adulthood. Our study further contributes to previous work exploring associations between physical fitness and selective attention performed on this age group.

Thus, the aim of this study was to investigate if physical fitness was associated with cognitive function in healthy male high-school students. To do this, participants completed a $\overset{\cdot }{\text{V}}{\text{O}}_{2\max}$ test, answered questions regarding weekly physical activity, underwent a body composition test and performed a pc-based visuospatial attention task to determine performance in the executive domains of selective attention and inhibitory control. The Posner cue target paradigm (Posner, 1980) was chosen to study the association of cardiorespiratory fitness and attention capacity to stimuli with different attentional load. Responding to stimuli presented after an invalid cue is particularly demanding, compared to stimuli following valid cue and no cue presentations, as it involves an interaction between goal-directed actions and inhibition of reflexive bottom-up processing of attention initiated by the distracting cue. To have a homogeneous population, only males were included. The main hypothesis was that higher physical fitness is associated with faster reaction time (RT) for all stimuli categories, due to better selective attention and inhibitory control. Moreover, we expected to find the fastest RT for stimuli following valid cue, followed by invalid cue and no cue.

## Materials and Methods

### Participants

Out of the 85 young male students attending two different high schools in Norway, 66 (77.7%) were recruited, with 54 (63.5%) participants (17.9 ± 0.9 years, 72 ± 11 kg and 182 ± 7 cm) completing both the physical test and cognitive task.

### Procedures

Information regarding the study was sent via e-mail to the principals of two different high schools in Norway, and they replied quickly, expressing their willingness to participate in the study. An information meeting was then arranged at which the students who were interested in participating in the study, signed informed consent forms. When recruiting participants, it was made clear that both trained/active and untrained/sedentary participants were needed.

**Figure 1:**
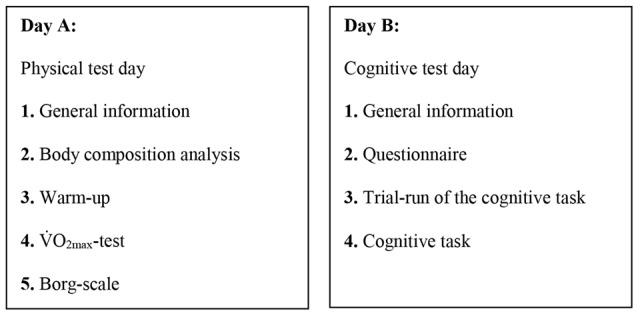
Test-sequence of day A and day B.

Data was collected in the course of 6 weeks, from September–November 2015. The tests were conducted in two independent test-runs for all participants, one for the physical tests (Day A) and one for the cognitive task (Day B; Figure 1). The cognitive task was performed between 10:15 and 15:22. On average, there was 12 ± 10 days between tests. All physical tests were conducted in the physiological test lab at Bergen University College, and the cognitive task was mainly completed at the students’ respective schools. Five to ten minutes before the participants started with the cognitive performance task, they completed a web-based electronic questionnaire.

All participants were told to dress appropriately for the physical tests, wearing shorts or pants, a t-shirt and running shoes. Participants were asked if they had felt ill during the last week before the test, especially with symptoms of fever and/or impaired general condition. Participants were excluded if they had been diagnosed with heart disease, asthma or any type of disease that could potentially affect their cardiorespiratory system in a strenuous exercise test. Before testing, all participants were informed orally of the test procedures and were given the opportunity to ask questions regarding the tests. In addition, they were asked not to engage in any strenuous physical exercise the day before the physical test as this could affect the results.

### Body Composition

A direct segmental multi-frequency bioelectrical impedance analysis (DSM-BIA) for determining body composition (body weight (kg), muscle mass (%)) was performed using the In-Body720 (Biospace Co. Ltd, Seoul, Korea) body composition analyzer (Lim et al., 2009; Tompuri et al., 2015). Standard procedures were followed for all participants.

### Cardiorespiratory Fitness Test

The test started with a 10-min warm-up with a gradient of 1.7% on a motorized treadmill (Woodway PPS 55, USA) where the velocity was gradually increased until the participant maintained a heart rate (HR) in the area of 73%–82% of age-predicted maximum HR (HR_max_). Following a short rest period (<3 min), an incremental test protocol was followed with a treadmill-gradient of 5.3% and a start velocity of 8 km · t^−1^. The manual test protocol was controlled by the test leader, whom increased the speed by 1 km · t^−1^ each minute until volitional exhaustion. Notifications of the amount of time left to complete each speed and the amount of time left until the apparatus would attain a new sample registration were given throughout. When each participant was nearing possible exhaustion (respiratory exchange ratio >1), the test leader asked if a speed increase would be tolerable; the participant then had the opportunity, through the use of different hand signals, to agree to a velocity increase of 1 km · t^−1^ or 0.5 km · t^−1^, or to maintain the same speed.

Oxygen uptake was measured using a computer that was connected to a metabolic system with a 4.2-Liter (L) mixing chamber, containing baffles (Oxycon Pro, Erich Jaeger GmbH, Hoechberg, Germany). A full system calibration was performed before the testing of each group of participants, and a volume calibration using a 3 L calibration syringe with an accuracy of 1/2 ± of 1% (Hans Rudolph Inc., Shawnee, KS, USA) was regularly performed between and during the testing of each group. Gas calibration was performed using a container that held 300 L of a gas mixture (Riessner-Gase GmbH, Carefusion, Germany) with a mixture ratio of carbon dioxide (5.84 vol. %), oxygen (15.00 vol. %), nitrogen (79.16 vol. %). Measurements of oxygen uptake were continuously recorded and saved at 30-s intervals. HR was monitored every fifth seconds using the RS400 (Polar, Kempele, Finland).

The average value of the two highest consecutive $\overset{\cdot }{\text{V}}{\text{O}}_{\text{2}}$ values (30-s intervals) for each participant, in any interval registration, was recorded as their individual $\overset{\cdot }{\text{V}}{\text{O}}_{2\max}$. $\overset{\cdot }{\text{V}}{\text{O}}_{2\max}$ was defined when two of three criteria were satisfied (Dupuy et al., 2015): (1) attaining a $\overset{\cdot }{\text{V}}{\text{O}}_{\text{2}}$-plateau; (2) attaining a HR >90% or equivalent to their age-predicted maximum (i.e., 220 minus age); and (3) attaining a respiratory exchange ratio >1.1.

### Cognitive Performance Task

**Figure 2:**
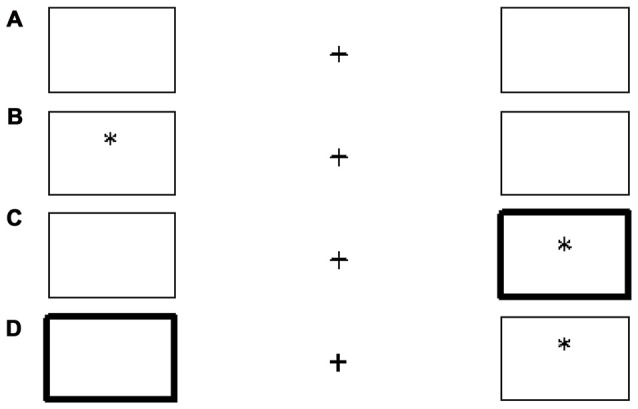
Cognitive performance task. **(A)** Standard output screen display with a crosshair and two horizontal rectangles. **(B)** Target stimulus appears without cue (no cue). **(C)** Target stimulus appears following a cue (valid cue). **(D)** Target stimulus appears opposite to cue rectangle (invalid cue).

A visual cognitive task based on the Posner cue paradigm was used to assess response time (RT), accuracy and inhibition (Posner, 1980; Posner and Cohen, 1984; Gundersen et al., 2007; Irgens-Hansen et al., 2015; Figure 2). The task was performed on a 13.3^″^ laptop, and was programmed with E-prime 2.0, standard version (Psychology Software Tools, Inc.).

The participants were instructed to fixate on the crosshair in the middle of the screen (Figure 2A), and to respond as fast as they could by pressing “l” on the keyboard when the target stimulus (an asterisk) appeared in the right rectangle and “d” when the target stimulus appeared in the left rectangle. Sometimes during the task, the frame on one of the rectangles became broader (a cue) before the target stimulus appeared. Participants were told to ignore the cue stimulus and only press the key when the target stimulus appeared.

All participants were instructed orally and performed a small practice run before the actual task to make sure they had understood the procedure. The task duration was 9 min and 20 s, and it was carried out in a quiet location without distractions (usually a small classroom), wearing hearing protectors to exclude distracting noise.

Three different stimuli categories appeared in the task: “no cue”, “valid cue” and “invalid cue”. If the target stimuli appeared in one of the rectangles without a cue, this was called a no cue presentation (Figure 2B). If the target stimulus appeared within the rectangle with a broader frame, it was called a valid cue presentation (Figure 2C). The third category applied when the target stimulus appeared in the opposite rectangle to the cue location, and was called an invalid cue presentation (Figure 2D).

The task consisted of 336 target stimuli, with each stimulus being presented for 500 ms with an interstimuli-interval (time between stimulus) of between 600 ms and 1400 ms. The inter-stimuli interval was selected from a set of fixed values (600, 700, 900, 1000 and 1200 ms). The order of the inter-stimuli interval, stimuli and cues was fixed for all participants, with mean interstimuli-interval of 825 ± 253 ms for no cue stimuli, 1108 ± 112 ms for valid stimuli and 1164 ± 236 for invalid stimuli presentations. The cue appeared in 50% of the cases 200 ms before the target stimuli, and in 50% of the cases 400 ms before the target stimulus would appear. Fifty-six (17%) of the target stimuli would appear without a cue (no cue), 224 (67%) would appear in the same rectangle as the cue (valid cue), and 56 (17%) would appear in the rectangle opposite to the cue (invalid cue).

RT (i.e., processing speed) and response accuracy were recorded and stored on the laptop for each trial. Mean RT for stimuli following no cue, valid cue and invalid cue were calculated for each participant. Error rate for the different stimuli categories were presented in percentage. Registration of responses before the target stimulus appeared, and responses during the first 149 ms. after target stimuli presentation were defined as erroneous responses (Amano et al., 2006). If an erroneous response was corrected by pressing a second time before the next stimulus was presented, it was still considered an erroneous response.

### Questionnaire

The questionnaire included questions about weekly physical activity (1 = less than once a week, 2 = 1–2 time a week, 3 = 3–4 times a week, 4 = 5–6 times a week, 5 = 7–8 times a week, 6 = 9–10 times a week), and variables that previously have been associated with cognitive performance in visuospatial tests, like ADHD diagnosis (yes/no; McDonald et al., 1999), dyslexia (yes/no; Franceschini et al., 2012), daily nicotine use (yes/no; Heishman et al., 2010) and daily video-game playing (minutes; Green and Bavelier, 2003). In order to calculate the potential covariate effects of alertness on the association of cardiorespiratory fitness and cognitive performance, the participants were also asked to rate their present alertness level on a 5-point Likert scale, ranging from 1 (not alert at all) to 5 (highly alert).

#### Ethics

The study was conducted in accordance with the Helsinki declaration. All participants were informed about the study and signed an informed consent form before participating. Participants could withdraw from the study at any point, and results were treated anonymously. Norwegian Social Science Data Services (NSD) approved the study (reference number: 44551).

### Data Analysis

The descriptive data is shown as range, mean ± standard deviation (SD). The data was checked for outliers and the preliminary analysis ensured no violation of the assumptions of normality.

To investigate if physical fitness affected RT differently for stimuli following no cue, valid cue and invalid cue, a linear mixed model was applied. Stimuli (no cue, valid cue and invalid cue) were used as repeated measure and subject as random intercept. Main effect of RT for the different stimuli categories, and interactions between RT for the different stimuli and $\overset{\cdot }{\text{V}}{\text{O}}_{2\max}$, muscle mass and weekly training were included in the model. Furthermore, the model was adjusted for daily video-game playing, time-point of cognitive testing and alertness. *Post hoc* test with Bonferroni correction was performed to evaluate differences in RT between the different stimuli. A model with a first autoregressive (AR1) covariance structure and maximum likelihood of 10,000 iterations was applied showing an acceptable model fit (AIC = 1454).

Because no participants reported being diagnosed with ADHD, only three participants reported dyslexia and seven reported daily nicotine use, those variables were not included in the regression analyses. For all statistical analyses, *p*-values of <0.05 were considered statistically significant. SPSS® version 23.0 (IBM Corporation, Armonk, NY, USA) for Windows® was used for all the statistical analyses.

## Results

A significant main effect of stimuli (*F* = 6.01, *p* = 0.003) was found. Faster RT was seen on stimuli following valid cue (Estimate = −146.89, SE = 43.42, *p* = 0.001), but not on stimuli following no cue (Estimate = −65.62, SE = 32.96, *p* = 0.051) when invalid cue was the reference. However, *post hoc* analyses with Bonferroni correction showed significant differences between all stimuli categories. RT to stimuli following invalid cues were slower compared to valid cue (Mean differences = 59.94, SE = 2.81, *p* < 0.001) and faster compared to stimuli following no cue (Mean differences = −23.16, SE = 2.13, *p* < 0.001). RT to valid cue was faster compared to no cue (Mean differences = −83.10, SE = 2.8, *p* < 0.001). For mean values, see Table 1.

**Table 1:** An overview of scores from the physical, cognitive and questionnaire assessments of the participants (*n* = 54).

	Mean ± SD	Range
RT (ms)		
Stimuli after no cues	376 ± 31	313–451
Stimuli after valid cues	293 ± 28	239–364
Stimuli after invalid cues	353 ± 35	278–429
Errors (%)		
Stimuli after no cues	3.3 ± 3.0	0–14.3
Stimuli after valid cues	5.9 ± 5.0	0.5–20.5
Stimuli after invalid cues	10.0 ± 7.6	0–32.1
$\overset{\cdot }{\text{V}}{\text{O}}_{\text{2}}$ max (ml/min/kg)	54.2 ± 4.9	41.9–66.7
Muscle mass (%)	49.8 ± 3.3	40.4–54.5
Weekly training sessions:		
Less than once a week	9%	
1–2	24%	
3–4	33%	
5–6	19%	
7–8	13%	
9–10	2%	
Daily video game playing (min)	73 ± 67	0–360
Time-point of cognitive testing	11:50 ± 1.0	10:15–15:22
Alertness	3.5 ± 0.8	1–5

*Data is presented as mean ± standard deviation (SD) and range (min-max)*.

**Figure 3:**
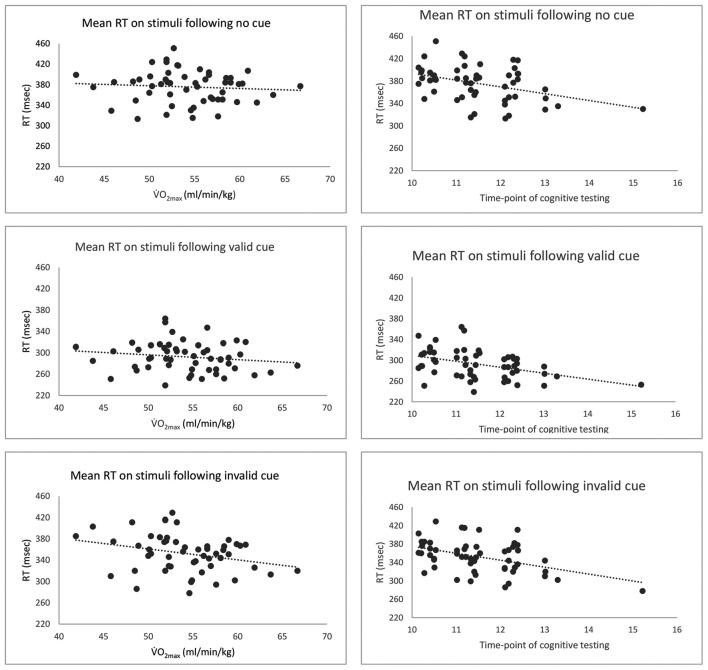
An overview of correlations between $\overset{\cdot }{\text{V}}{\text{O}}_{2\max}$ (ml/min/kg) and time-point of cognitive testing, and mean reaction time (RT; ms) to stimuli following no cue, valid cue and invalid cue for each participant. The dotted lines represent the linear trend.

Moreover, there was a significant interaction of $\overset{\cdot }{\text{V}}{\text{O}}_{2\max}$ and stimuli (*F* = 3.67, *p* = 0.017). Higher $\overset{\cdot }{\text{V}}{\text{O}}_{2\max}$ was associated with faster RT for stimuli following invalid cue (Estimate = −2.69, SE = 1.03, *p* = 0.011), and for stimuli following valid cue (Estimate = −2.08, SE = 1.03, *p* = 0.048), but not for stimuli following no cue (Estimate = −1.16, SE = 1.03, *p* = 0.266). There was no interaction of muscle mass and stimuli (*F* = 1.01, *p* = 0.397) or of weekly training and stimuli (*F* = 0.99, *p* = 0.405). Time-point of cognitive testing was the only covariate that contributed significantly to the model (*F* = 14.14, *p* < 0.001) with significantly slower RT in the morning (Estimate = −12.32, SE = 3.28, *p* < 0.001). See Figure 3 for an overview of correlations between RTs of the different stimuli and $\overset{\cdot }{\text{V}}{\text{O}}_{2\max}$ and for the RTs of the different stimuli and the time-point of cognitive testing.

## Discussion

Our results showed a significant interaction of $\overset{\cdot }{\text{V}}{\text{O}}_{2\max}$ and RTs for the different stimuli categories. The interaction was significant for stimuli following valid and invalid cues, with an association between higher $\overset{\cdot }{\text{V}}{\text{O}}_{2\max}$ and faster RT. There was also a significant association between time-point of cognitive testing and RT, with slower RT in the morning.

The findings of the present study show an association between higher $\overset{\cdot }{\text{V}}{\text{O}}_{2\max}$ and faster RT for stimuli following both valid and invalid cues. Both stimuli conditions initiate a reflexive bottom-up attraction of attention due to the appearing peripheral cue, but differ in their demand as the most often appearing valid cue (67%) does not demand inhibition. The invalid condition (17%) will therefore demand more of a volitional top-down control when having to inhibit the most probable response. Higher $\overset{\cdot }{\text{V}}{\text{O}}_{2\max}$ seems to be beneficial in conditions initiated by cues through a faster processing speed of information, indicating a relationship between cardiorespiratory fitness and selective attention and inhibitory control in conditions where facilitation occurs.

Our findings are in agreement with a previous study by Themanson et al. (2008) showing that higher fit individuals exhibited a better top-down control of attention, and a better modulation of responses to tasks measuring selective attention. In addition, our results support previous studies showing that physical fitness yield favorable effects on executive functions (Gomez-Pinilla and Hillman, 2013; Erickson et al., 2014; Dupuy et al., 2015; Guiney et al., 2015; Szuhany et al., 2015; Luque-Casado et al., 2016). No interaction between $\overset{\cdot }{\text{V}}{\text{O}}_{2\max}$ and RT on stimuli following no cue in the present study may be due to a lower attentional demand, or to the lack of priming in that stimuli category.

Our results showed fastest RT for stimuli following valid cue, followed by invalid cue and no cue. As our results show, cueing was consistently associated with faster RT, possibly reflecting a higher internal activation and increased responsiveness to upcoming target stimulus caused by the priming effect (Mangun and Hillyard, 1991; Gabay and Henik, 2008; Hayward and Ristic, 2013). Lack of priming may explain the slowest RT for stimuli following no cue. Slowest RT for no cue could also be related to the shorter interstimuli-interval. Time-point for cognitive testing were associated with RT with highest RT in the morning, supporting common knowledge of the circadian variation in alertness and performance (Van Dongen and Dinges, 2010).

$\overset{\cdot }{\text{V}}{\text{O}}_{2\max}$ was the only significant physical fitness-predictor of cognitive performance in the present study. Our finding is in accordance with previous studies showing that cardiorespiratory fitness is regarded as one of the components of physical fitness mostly associated with cognitive performance (Ruiz-Ariza et al., 2017).

It is worth noting that there are recent studies failing to demonstrate the fitness-executive function hypothesis (Verburgh et al., 2014; Ballester et al., 2015). Moreover, Belsky et al. (2015) found that children with higher IQ scores grew up to be adults who were less sedentary and less obese, and in turn, had better cardiorespiratory fitness. They suggest that socioeconomic status, household and neuroselection must be considered when investigating the fitness-executive function hypothesis. Thus, more studies including other explanatory factors is needed.

Participants in the present study demonstrated an above average cardiorespiratory fitness ($\overset{\cdot }{\text{V}}{\text{O}}_{2\max}$ score of 54.2 ± 4.9 mL · kg^−1^ · min^−1^) compared to the age-relative norm of the population (46–50 mL · kg^−1^ · min^−1^; Shvartz and Reibold, 1990). Thus, we cannot exclude a greater association of $\overset{\cdot }{\text{V}}{\text{O}}_{2\max}$ and cognitive function in participants whose mean score is closer to the population average, given normally distributed $\overset{\cdot }{\text{V}}{\text{O}}_{2\max}$ scores.

### Limitations and Strengths

Due to the general limitations of a cross-sectional design, it is impossible to establish cause and effect in this study. In addition, the above average fitness of the participants has some implications for generalizability. The fact that the study exclusively recruited males and that the sample size is relatively small contributes to this generalization problem. Moreover, the spatial visual attention task used in the present study may not be appropriate to identify whether the cueing effects are due to endogenous spatial orienting driven by probability manipulation, attentional capture (peripheral cues), stimulus-response compatibility, or a mixture of all of them. The strengths of the study are the use of direct measures of $\overset{\cdot }{\text{V}}{\text{O}}_{2\max}$ and a pc-based cognitive performance task with different attentional loads and the homogeneous group regarding age and gender.

## Conclusion

The findings of this study show that cardiorespiratory fitness is associated with cognitive performance in healthy male high-school students. Our results suggest that cardiorespiratory fitness may be associated with better modulation of bottom-up and top-down processes in tasks involving selective attention. More research is needed to increase the knowledge about factors affecting cognitive function.

### Implications for Future Research

This study contributes to the limited number of studies investigating the influence of physical fitness on the components of selective attention and inhibition in male high-school students. More studies are needed to further assess the association of physical fitness and attentional tasks demanding inhibitory control, and how this relates to the ability to maintain attention.

## Author Contributions

All authors participated in planning the experiment. EW, MK and HG participated in data collection. All authors have participated in conception of this manuscript, and have assisted in revising the manuscript for important content. EW and HG wrote the first draft of the manuscript, and all authors have provided final approval of the enclosed manuscript.

## Conflict of Interest Statement

The authors declare that the research was conducted in the absence of any commercial or financial relationships that could be construed as a potential conflict of interest.
